# Building a new life: a qualitative study of how family carers deal with significant changes

**DOI:** 10.1186/s12877-022-03236-8

**Published:** 2022-07-01

**Authors:** Wendy Duggleby, Hannah M. O’Rourke, Pamela Baxter, Cheryl Nekolaichuk, Genevieve Thompson, Shelley Peacock, Sunita Ghosh, Jayna Holroyd-Leduc, Carrie McAiney, Véronique Dubé, Jennifer Swindle, Madeleine Pagnucco-Renaud, Samina Sana

**Affiliations:** 1 Faculty of Nursing University of Alberta, 4-141 ECHA, 11405 87th Ave, Edmonton, AB T6G 1C9 Canada; 2 Faculty of Health Sciences, McMaster University, 1280 Main St. W, Hamilton, ON L8S4K1 Canada; 3 Department of Oncology, Faculty of Medicine and Dentistry, University of Alberta, Edmonton, AB T6L 0A3 Canada; 4 College of Nursing, University of Manitoba, 89 Curry Place, Winnipeg, MB R3T 2N2 Canada; 5 College of Nursing, University of Saskatchewan, 104 Clinic Place, Saskatoon, SK S7N 2Z4 Canada; 6 Alberta Health Services-Cancer Control Alberta, Department of Medical Oncology, University of Alberta, 11560 University Ave, Edmonton, AB T6G 1Z2 Canada; 7 Department of Medicine, Cumming School of Medicine, University of Calgary, 1403 29th Street NW, Calgary, AB T2N 4W4 Canada; 8 Schlegel Research Chair in Dementia, Schlegel-UW Research Institute for Aging, Waterloo, Canada; 9 School of Public Health and Health Systems, University of Waterloo, 200 University Ave, W, Waterloo, ON N2L 3G1 Canada; 10 Chairholder Marguerite-d’Youville Research Chair, Faculty of Nursing, University of Montreal, P.O. Box 6128, Centre-ville Station, Montreal, QC H3C 3J7 Canada; 11 Faculty of Nursing, University of Alberta, Level 3 ECHA, 11405 87 Avenue, Edmonton, Alberta T6G 1C9 Canada; 12 Faculty of Nursing University of Alberta, 4-005A, ECHA, 11405 87th Ave, Edmonton, AB T6G 1C9 Canada; 13 Faculty of Nursing, School of Public Health, University of Alberta, Edmonton, Canada

**Keywords:** Family caregiving, Nursing homes, Dementia, Qualitative

## Abstract

**Background:**

Family carers of persons living with dementia who are residing in long term care (LTC), often experience significant changes in their roles and relationships which affects mental and physical health. Research has focused on describing the carers’ experience, but not on how they deal with these changes or their perceptions of support needs. The purpose of this study was to explore how family carers of persons living with dementia residing in LTC deal with significant changes and to understand how best to support these carers.

**Methods:**

Eight face-to-face audio-recorded focus group interviews were conducted with 45 participants from September 2019 to January 2020, as part of a larger study aimed at guiding the adaptation of an online toolkit to support family carers of persons living with dementia residing in LTC. Applied thematic analysis was used to analyze the focus group data.

**Findings/results:**

Carers dealt with the significant changes they experienced through the process of “building a new life” consisting of two sub-processes: a) building new relationships (with their family member, LTC staff and others outside of LTC), and b) finding space for themselves (sharing of care and finding balance). Understanding dementia, support from others (staff, family and friends), connecting with resources, and being included in care decisions helped carers build a new life.

**Conclusion:**

The process of building a new life describes the ways that family carers deal with the life-altering changes they experienced when a family member is admitted to LTC. Carers may be supported in building their new life, by providing them with information about dementia and how to relate to staff and their family member living with dementia. The quality of care being provided and the LTC environment may also play an important role in how carers deal with the significant changes they experience.

## Background

Worldwide, family carers are the critical sources of support for persons living with dementia [1]. Family carers of persons living with dementia are relatives, friends, or neighbors who usually provide unpaid care and continue to provide care even after their relative moves into long-term care (LTC) [2, 3]. Carers may experience relief when their family member is admitted to LTC, but several studies have shown that they often continue to experience burden and emotional distress [4–7]. These feelings may be due to their negative perceptions of dementia-care and feeling that they have abandoned their family member [8, 9]. Family carers of persons living with dementia residing in LTC are at-risk for poor mental health outcomes as their mental health often worsens after their family member moves into LTC and need ongoing support [10, 11].

A narrative review of 12 qualitative studies describing the experiences of carers of persons living with dementia residing in LTC clearly identified the worry and anxiety, grief and loss, guilt and despair experienced by these carers [12]. Hennings and Froggatt [8] also described the significant changes that carers experienced such as the evolving relationships with their family member, their own need for companionship and the need to face end-of-life issues. More recent studies suggest that carers of persons residing in LTC also experience significant changes such as: a transfer in responsibilities from being the primary caregiver to a visitor, new relationships with LTC staff members, and feelings of grief and loss of hope [11, 13, 14]. Carers need to adjust to a new way of living both for themselves and for the person with dementia [10, 15]. However, the literature remains unclear as to the best ways to support family carers as two recent systematic reviews suggested there is little evidence available, and there is a significant need to improve evidence-based interventions to support carers [16, 17].

### Purpose

The purpose of this study was to address the following research questions: 1) How do carers deal with significant changes when their family member with dementia resides in LTC? and 2) What are the carers’ perceptions of their support needs when dealing with these changes?

## Methods

This qualitative study was part of a larger study whose purpose was to adapt and evaluate an online toolkit (My Tools 4 Care-In Care [MT4C-In Care]) to support family carers of persons living with dementia in LTC [18]. In the first phase of the larger study focus groups were conducted with carers of persons living with dementia in LTC. Focus groups were utilized as they have been found to elicit more personal, sensitive data than individual interviews [19]. The focus groups addressed the following questions: What is the experience of significant changes for carers of persons living with dementia in LTC facilities? What would help with these changes?; What are carers’ experience of loneliness?; and How should MT4C-In Care be adapted to best suit carers’ needs? This article reports findings related to the significant changes that carers experiences through an analysis of data gathered from the first three questions. A discussion of factors associated with carers’ feelings of loneliness and how MT4C-In Care should be adapted to address loneliness is reported elsewhere (O'Rourke at al in preparation Adapting a Psycho- educational Intervention to Address Loneliness of Informal Carers).

The qualitative focus group data were analyzed using an applied qualitative thematic approach [20]. This includes aspects of grounded theory and phenomenology to address research questions in applied research settings. Using this approach allowed the research team to identify and describe processes of how carers dealt with the significant changes they experience without developing a substantive theory that is often the outcome of grounded theory. The multidisciplinary research team included nurses, a geriatrician, a statistician, and a clinical psychologist who contributed to aspects of the study. Focus group interview data were collected from September 2019 to January 2020.

Ethics approvals for these interviews involving human participants were successfully obtained in all involved provinces (Alberta, Pro00090771; Saskatchewan, 1385; Manitoba, E2019:09; Ontario, 7659). The Standards for Reporting Qualitative Research (SRQR) guidelines [21] were incorporated into this report.

### Participants and setting

There were multiple community and LTC settings for this study, as it was conducted in four Canadian provinces (Alberta, Saskatchewan, Manitoba, and Ontario). Forty-five participants were recruited using convenience sampling through different provincial Alzheimer Societies and LTC homes. Inclusion criterion were: 1) > 18 years of age, 2) English speaking, 3) self-identified as a family/friend of older (> 65 years of age) persons in any stage of dementia currently residing in LTC, and 4) a valid email address and access to a working computer and internet. Potential participants who met the inclusion criteria were asked by staff of the recruiting organizations if they were interested in participating in a focus group. With their permission the email addresses of potential participants were sent to trained research assistants. The research assistants then contacted potential participants, explained the study and answered any questions.

### Data collection

In advance of the focus group sessions, a copy of the demographic form, focus group questions and consent forms were emailed to the participants. At the start of the focus group session, research assistants obtained written informed consent and collected demographic information using a self- report form (age, sex, marital status, ethnicity, citizenship, language, education, employment, medical conditions, religion, annual income, and relationship to the care receiver). Carer participants were given information about the different stages of dementia and asked to determine their family member stage of dementia.

Two face-to-face focus group interviews were conducted in each of four provinces by two trained facilitators (eight focus groups in total). One facilitator led the session (moderator), while the other took detailed notes. Three to eight carers participated in an approximately 90-minute audio-taped focus groups. Focus groups were conducted in locations that were comfortable and easily accessible for the participants. Moderators used a semi structured interview guide and asked the following questions:

- Could you please tell us about the biggest changes you have experienced as a caregiver after your family member and/or friend was admitted to a long-term-care facility?
- What would help with these changes?
- Can you tell us about a time when you felt left out or isolated after your family member/friend was living in long-term care?

Moderators adapted the questions based on the responses of the participants and also used prompts and follow up questions to gather more in-depth information.

### Data analysis

Demographic information was entered into SPSSv24 [22] and analyzed using descriptive statistics. All interview data and field notes were transcribed and anonymized by an experienced transcriptionist and checked for accuracy by research assistants in each province. Focus group data were aggregated from all sites and analyzed together. Guest et al.’s [20] applied thematic analysis was conducted using the following steps: 1) transcripts were read and reread as a whole with a focus of the analytic objective (identifying processes), 2) common patterns in the data across groups were identified examining similarities and differences 3) data were grouped into processes using a constant comparative approach and 4) collective high level themes representing all the focus group data were identified. Then based on these high level themes, an overarching theme was conceptualized. The first four steps were completed by the first author, then the research team reviewed the findings and data. Findings were revised until agreement within the research team was reached. The participants own words were used as much as possible to describe the themes and an audit trail was kept to ensure trustworthiness of the data [20].

### Findings

#### Participants

Table 1 describes the characteristics of the 45 participants, who had a mean age of 65.16 years (SD = 10.96) and a median age of 65 years. Focus group participants were predominantly female (31/45; 68.9%) and married (29/45;64.4%). The majority self-identified as Caucasian (42/45.93.3%) and spoke English (43/45;95.5%) as their first language. Most were not employed (31/45, 68.9%),were spouses/partners (22/45, 48.9%) Overall, 42.2% (19/45) of participants said they themselves had a medical condition, with a variety of conditions being reported such as anxiety, depression, cardiovascular disease, cancer, and chronic kidney disease. The majority of participants reported their finances met their need: 17 participants (17/45.37.8%) reported it met them completely, and 15 (15/45. 33.3%) reported they were met adequately.

**Table 1:** Focus group participant demographic characteristics (*n* = 45)

Variable	Mean (SD)	Range
Age (years)	65.16 (10.96)	30–85
Education (years)	15.16 (3.42)	3.5–23
	N	%
Sex		
Female	31	68.9
Male	14	31.1
Marital Status		
Single	10	22.2
Married	29	64.4
Widowed	1	2.2
Divorced/Separated	3	6.7
Ethnicity		
Caucasian	42	93.3
Other	3	6.7
Primary Language		
English	43	95.5
French	2	4.4
Employed	14	31.1
Not Employed	31	68.9
Relationship		
Spouse/Partners	22	48.9
Children	18	40
Medical Conditions		
Yes	19	42.2
No	26	57.7
Finances Met their Needs		
Completely	17	37.8
Adequately	15	33.3
Did not meet their needs	4	8.9
Missing	9	20

Table 2 describes the characteristics of the care recipients residing in LTC who had an average age of 80.09 years old (SD = 11.42) and had resided in LTC on average of approximately 2 years (range 1 to 180 months). The majority were female (30/45, 66.7%). The care recipients were at different stages of dementia ( [23], with 46.7% (21/45) in late stage, 17.8% (8/45) middle stage, four were end of life (4/45.8.9%) and middle/late stage (4/45.8.9%) and one was in early stage of disease (1/45, 2.2%).

**Table 2:** Care recipient demographic characteristics

Variable	Mean (SD)	Range
Age	80.09 (11.42)	67–104 years
Months Since Long-term Care Admission	23.72 (28.92)	1–180 months
	N	%
Sex		
Female	30	66.7%
Male	15	33.3%
Stage of Disease		
Early stage	1	2.2%
Middle stage	8	17.8%
Mid−/Late stage	4	8.9%
Late stage	21	46.7%
End-of-life	4	8.9%
Missing	7	15.6%

### Building a new life

Participants dealt with the significant changes (such as changes in roles and relationships and their physical and mental health) by “Building a New Life”. Some of the participants felt they had to build this new life alone. As one participant said “Building a new life- alone. You have to establish ... your new life” (FG3). Another participant said that “…. this is a different life for now. And temporary….” (FG6) suggesting this new life is one they realized would change as their family member’s condition progressed. Participants noted benefits to building this new life such as being able to reconnect with activities and others they had once enjoyed: “….and I’m finding that I’ve started new things. I get back doing things that I enjoy, things that I’ve had to put off quite a while” (FG 4).

**Fig. 1:**
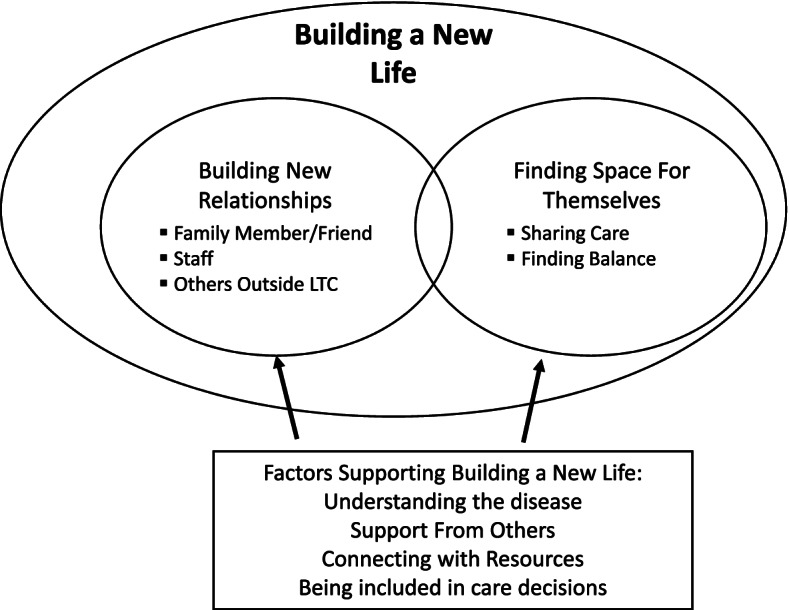
Building a New life

They built a new life by: a) building new relationships (with their family member/friend, with LTC staff and with others outside of LTC), and b) finding space for themselves (described in detail below). Factors supporting building a new life were: a) understanding dementia, b) support from others, c) connecting with resources, and d) being included in care decisions. Figure 1 illustrates these findings and Table 3 presents transcripts examples for the themes and subthemes.

**Table 3:** Building a new life themes, sub themes and data excerpts

Theme/Sub Theme	Data Excerpts
***Building New Relationships***	“The change in your relationship with your loved one or the changes in your other relationships that you may be having on a day-to-day basis” (FG9).
/With Family Member	“I categorize most of the changes as having to do with building a new relationship [with my family member living in LTC].”” (FG3)
/People Outside of LTC	“So, I see it in one hand a relationship with your spouse and on the other hand building a new relationship outside of that relationship” (FG3)
/With Staff	“Knowing the staff, the PSWs [personal support workers], they’re your link” (FG7).
***Finding Space for Themselves***	“…this inside pulling of trying to be loving to your mother but also trying to find that space for yourself. “(FG8)
/Sharing Care	“So, you know, you’ve given up the day-to-day physical. Now, there’s the emotional and the worry, right? So, it’s different” (FG1).
/Finding Balance	“So, what happens is like I work till late at night trying to get laundry done and things like that. And in the morning, I’m tired because just to – and it’s hard to get a balance” (FG7)
***Factors Supporting Building a New Life:***	
/Understanding Dementia	“I think it’s understanding the disease.” (FG7)
/Support from Others	“Calling on others for support, I think, is huge. That’s one of the things that I said, you know, just rallying the troops”(FG8)
/Connecting with Resources	“As well as maybe a resource list of when things kind of fail, you can reach out to these people to help you advocate for what they need” (FG3).
/Being Included in Care Decisions	“And we’re not getting care conferences. …I think that’s a way of isolating me, like keeping me out of the loop” FG3)

#### Building new relationships

New relationships were created with their family member/friend residing in LTC, with LTC staff and with others outside of LTC. These subthemes are described below.

*Family Member/Friend*; The new relationships with their family member with dementia was attributed to the decline in their family member’s health:

I categorize most of the changes as having to do with building a new relationship [with my family member living in LTC]. It’s not something that happened the day they moved into long-term care because this has been already quite a long journey in terms of seeing a decline at home and caring at home and then getting personal care at home and home care in. So, there has been stages all along the way (FG3).

Participants described the changes with their family members as occurring over time. One of the most distressing changes was related to the declining ability of their family member to communicate:

It’s a lack of communication. Sorry. As my mom when she moved into the long [term] care facility, the hill that’s she’s going downhill is steeper, quicker, and for a lack of her – because she wasn’t doing as much, it’s her lack of communication. I can’t sit and have a conversation with my mom. Which makes – feel lonely…. (FG1).

However, changes in relationships and the caregiving role were not always perceived as negative:

Because I do find that one of the major changes in my wife going to long-term care is that now I’m not the bad guy. I’m not the one making her have to take her medication. They are. So, when I go in there, she’s actually happy to see me again” (FG2).

*Staff:* Building relationships with staff was also important for the participants: “Knowing the staff, the PSWs [personal support workers], they’re your link” (FG7). As one participant explained: “You have to become part of the team but you can’t become in charge of the team. You have to just work with the team” (FG8). Becoming a member of the team enabled carers to find space for themselves in their new life.

*Outside of LTC*: Re-establishing relationships with previous individuals or establishing new relationships with people outside of LTC was important as well:

And so, trying to navigate differently in terms of re-establishing some relationships you left behind. Or, establishing new relationships or maybe getting re-involved in some things that you withdrew from while you were a caregiver at home. So, I see it in one hand a relationship with your spouse and on the other hand building a new relationship outside of that relationship. So, those are the two big changes or categories of changes I see (FG3).

#### Finding space for themselves

To find space for themselves in their new life participants described negotiating the sharing of care of their family member with LTC staff and finding the right balance between their life with their family member in LTC and a life outside of LTC:

I’m not there all the time. So, when I’m home I feel like I should be there. But when I’m there I feel like I should be home. …. but you know, it’s really – it’s yeah, it’s this pull – this pulling – this inside pulling of trying to be loving to your mother but also trying to find that space for yourself. (FG8)

This quote suggests that it was not always easy for carers to find space for themselves, but it was important. Part of what made finding space for themselves difficult was the need to relinquish some of the care they had been providing to LTC staff.

#### Sharing care

Relinquishing some of the care of their family member to LTC staff in order to share the care of their relative/friend was difficult for some participants. For some it took time to realize they were not alone in providing care: “I’m not doing this by myself anymore.” (FG6). In another focus group participants described how they no longer provided hands on care, but provided emotional care for their family member instead. For example, one participant said:

Yeah, I think it’s a – I think - I mean, I’m sort of a caregiver but not as hands-on. So, when I see is– it’s a trade-off. So, you know, you’ve given up the day-to-day physical. Now, there’s the emotional and the worry, right? So, it’s different” (FG1).

However, in the same focus group a participant said that roles changed and the emotion and worry decreased over time:

I think that’s true at the beginning. When they’re admitted. But it will be two years for my wife next month. And you know, I think I don’t know how much we’re managing it now. It’s just the staff know her very well, sort of” (FG1).

Many participants were unable to engage in sharing of care because of their worry about the care being provided by the LTC staff. Participants expressed anger and frustration regarding the poor quality of care their family member received and ended up feeling they needed to be with their family member almost all of the time. As one participant said: “I have no more time to do anything. It is just like running around all the time. Total care” (FG7). This interfered with their ability to negotiate sharing of care with staff and subsequently find space for their new life.

The physical environment of the home at times also contributed to the carers’ feelings of guilt, worry and anxiety regarding their family member. For example, one participant said: “when my mom moved in to this facility, she was on the first floor and it was really dark. And it was just – it was – the physical environment was depressing”. (FG 7). As a result, she felt she needed to provide additional emotional support and care for her mother and visit more often then she would have if the environment was brighter. Sharing of care was also problematic if the food served in the facility was difficult to swallow or not appetizing. For some participants this meant they needed to bring food from home or attend each meal. The physical environment was also a factor that at times prevented participants from engaging in sharing of care.

#### Finding balance

Similar to sharing care, finding a balance between being with their family member in LTC and building a new life was difficult:

So, with me, if I come at least twice a day and I try to do three times a day. But it’s really hard. With, you know, like having a life, like doing your shopping and you know, shoveling your driveway and…(FG5).

This was particularly difficult when participants felt they were unable to relinquish some of the care to staff:

So, I try to come three times a day but when I come – and the other thing, is he doesn’t respond right away. They’ll leave him and I come in at 10:30 and he’s sitting in front of his breakfast so, it’s the added guilt of – you know (FG7).

Guilt was a feeling often described by participants when they were finding it difficult to balance their time with their family member and their life outside of LTC.

Finding a balance between their life with their family member and their outside life, also consisted of making decisions about their level of involvement in their family members care and when to intervene on their behalf:

… And you know, even um, decisions about how involved you get in the person’s care, like working out the relationship with the person that you’re caring for but also with the caregivers [staff] that are there and trying to find the right balance of intervention and so on (FG3).

### Factors supporting building a new life

What helped participants to build a new life was described in four themes: a) understanding dementia, b) support from others (staff, family and friends), c) connecting with resources, and d) being included in care decisions.

#### Understanding dementia

One participant described the importance of understanding dementia, as the disease had become part of them as well: “I think it’s understanding the disease. It’s not only with the patient. The disease is also with the caregiver” (FG7). This quote emphasized the interrelationships between the carer and their family member. This suggested that dementia is part of the carer’s life as well and understanding dementia would assist the carer to build a new life. Understanding dementia also helped participants prepare for the future:

…. she [physician] prepares you extremely well for the future. And once you adopt that, you can move forward, that’s what I found. If you try to fight it, you are in chaos the whole time. So, I think that in my case and I don’t know about others, but the more knowledge that I had and the more people that were experts, so-called, in their particular fields, whether it was O.T., physio, social worker, doctors who really understood dementia (FG 3).

Participants valued information about dementia from those they considered experts. They also emphasized that this knowledge was critical for preparing for their future and the future of their family member. Unfortunately, not all participants were able to access information from experts; “I don’t think that really good information is out there, either for people seeking it or for people that need to know” (FG2).

#### Support from others

Support from others, in particular other family members and friends, also helped:

Calling on others for support, I think, is huge. That’s one of the things that I said, you know, just rallying the troops. And just get everybody you know to come and visit [care recipient]. Come and visit for half an hour once a week or once a month. Or whatever you can afford, right, to take some of that pressure off (FG8).

Taking pressure off the carer to be physically present in the LTC home would also provide space to build a new life.

#### Connecting with resources

The participants also described the importance of resources to support them as carers: Something to help? …. so, I can discuss my concerns, can help build my confidence in the facility and the staff, as well as maybe a resource list of when things kind of fail, you can reach out to these people to help you advocate for what they need (FG3).

This quote suggested that resources were needed in several areas so the participant could advocate for a family member. Others also described wanting resources to reach out to when they were having a difficult time:

And support groups that would be great. I’d love to go to a support group but I had no idea there is such a thing. But just a general list of things in your community that you can reach out to and talk to and…” (FG8).

Participants also wanted to be able to talk with others carers of persons residing in LTC: “…. finding people who are in the same boat you are and finding out you’re going down the same river and paddling the same way, there’s nothing like it” (FG1). The opportunity to talk with other carers who were also having a similar experience was helpful as the participants often felt other people did not understand what they were going through.

#### Included in care decisions

Participants noted that being included in care decisions by the staff would also help with sharing of care:

And we’re not getting care conferences. …I think that’s a way of isolating me, like keeping me out of the loop. He has a lot of medical needs but I think – I get the feeling that the doctor and the nurses hub bub about and they don’t – you know, they don’t include me in the circle (FG3).

This feeling of exclusion regarding decision making about their family member was described in all of the focus groups. Some suggested it made them feel isolated which got worse as their family member’s condition worsened: “I felt isolated by the home…I felt they kind of took ownership of him and I sort of felt like I was left out of the decision-making… And it seems to get worse the – as his disease progresses” (FG3). Participants suggested monthly meetings may help:

I think one of the things I like the most with regards to that type of thing is that they have the monthly meeting with family members to deal with any concerns the family members have with regards to the care that their family members who are whether it’s the facility itself…(FG1).

A structured way of communicating with carers and provision of opportunities to be involved in care decision-making would support carers.

## Discussion

Carers adjusted to the significant changes they experienced when their family member resided in LTC by “Building a New Life”. It required them to seek help, as well as connect with resources and others while remaining involved in their family member’s care. Building a new life was achieved by building new relationships and finding space for themselves through sharing care and finding balance in their lives.

Building a new life, as a process described by carers to deal with the significant changes they experienced, is a unique finding in the literature. Although other studies have described carers of persons residing in LTC as having a new or different life [5, 10, 24], these studies did not describe the processes carers used to build this new life. As well they did not describe factors that support carers to build their new life.

The process of building a new life included building new relationships and finding space for themselves. In building their new life, participants described building new relationships with LTC staff. Building relationships with LTC staff was a common theme in several studies [10, 12, 25]. These studies described the challenges that carers face in building relationships with staff as staff perceived carers as ‘visitors’ who are difficult and demanding [10, 25]. Our findings suggested carers realized they needed to be a member of the team. To be a team member, carers worked to get to know the staff as individuals. This is similar to the findings of a qualitative study of seven carers, that suggested developing communication through informal contact with staff enabled family and staff alike to build a personal connection [25]. Carers highly valued interactions and communication with staff when they felt there was a personal connection [13]. Our findings extend these previous findings and suggested that establishing personal connections was an important component of communicating with LTC staff, but also with the goal of becoming a member of the care team.

Carers also had to continuously develop new relationships with their family member or friend with dementia. Some studies described difficulties and distress experienced by carers as a result of the physical deterioration and cognitive decline of their family member living with dementia [26, 27]. The literature also described changes in the carer’s role, as their tasks changed [5, 9, 15, 28]. However, these studies did not describe the work of carers to continuously redefine and develop new relationships with their family member that were described by our participants.

An important aspect of developing new relationships was the need for participants to develop relationships with others outside of the LTC setting. This seemed to help carers deal with feelings of loneliness which appears to be a common experience of carers of persons with dementia residing in LTC [10, 12]. Previous research has not described the need for carers to develop new relationships outside of LTC. Carers, particularly those who have limited social networks and are unable to share care, may find building new relationships outside of LTC difficult. In our study, developing new relationships outside of LTC was also an important step to building a new life. However, how best to support carers to develop new relationships requires further research.

The need for carers to find space for themselves has not been reported in previous studies. However, similar findings of sharing care and finding a balance have been reported. For example, relinquishing some aspects of care to LTC staff was described in several qualitative studies [9, 10, 15]. Many carers found this difficult, as they felt guilt and ongoing responsibility for their family members’ well-being [12, 28]. Our participants described difficulties related to relinquishing some of the care of their family member to LTC staff when they believed the quality of care was poor. When this occurred, balancing their two worlds, the one in LTC and the other outside of LTC was very difficult. The struggle of balancing two worlds, when carers were concerned about the care their family member was receiving, was also reported in a mixed methods study examining interpersonal and contextual factors that influence carer–staff relationships [13]. Although focused on carer-staff relationships, Zamora et al. (13)reported the quality and appropriateness of care was an important factor influencing carers’ experiences. Concerns were expressed regarding personal care provided, medication management, safety, falls, wandering, abuse and neglect resulting in carer distress and anxiety. Physical environment also impacted the carers’ experiences, as lack of privacy and room for activities worried the carers. In our study, it was the darkness of the environment and quality of the food that was most concerning. Thus, the quality of care and physical environment would possibly make it difficult for carers to feel they could share care and as a result be unable to find balance in their world with their family member in LTC and outside of LTC. However more research is needed to determine if this is the case.

In another study, finding a balance between their world within LTC and their world outside of LTC was key to carers finding hope in their lives [29], suggesting this balance important for carers’ mental health. However other studies did not describe how finding a balance and sharing of care was important for carers to find space for themselves as was described by our participants.

The participants described four aspects that helped them to build a new life: a) understanding dementia, b) support from others (staff, family and friends), c) connecting with resources, and d) being included in care decisions. Understanding dementia, support from others and connecting with information have also been described in previous studies. For example, the need for information and support is echoed in a study of 17 bereaved carers of persons with dementia who had resided in LTC [30]. Similar to our study, Thompson et al. [30] described informational needs of carers regarding dementia, its prognosis, its clinical course and end of life issues. However, in our study, the participants requested this information from those they considered experts. This is similar to findings from a meta-synthesis study of the experiences of carers of persons with advanced cancer [31]. In this study the authors synthesized the findings of 72 qualitative studies of carers’ experiences. They found timely information from trusted experts and networks supported carers during their experience. Similar to our findings and those of Thompson et al. (2020) supportive networks included family, friends and health care providers.

Connecting with resources involved carers having someone to express their concerns to, as well as resources to help them build confidence as an advocate for their family member. Like information, they wanted these resources to be available in a timely manner. These are similar findings reported in a recent systematic review of carers needs across settings [17]. In their review Mueller et al. [17], found carers wanted to improve their confidence in their new caregiving roles, such as communication with staff. They also wanted access to support groups and mental health services. In a qualitative study of 9 spousal carers in Australia suggested that peer support groups where carers can share their concerns, as well as navigator support to navigate the health and social care systems maybe useful resources for carers [32].

In previous literature, being included in care decisions has been seen as a benefit to residents and to carers as it increased their satisfaction with care [33]. For example, shared-decision making involving residents, families and LTC staff has benefited residents, such as improving residents’ care plans [33, 34]. The findings from our study suggests that being involved in care decisions has the additional benefit to the carers of decreased feelings of loneliness. Shared decision-making also helps carers with respect to relinquishing of care and moving to shared care, which facilitates the process of carers building a new life. However, more research is needed to verify if this is the case.

### Limitations

There were several limitations to this study, as most of the participants were white, well educated, female, married and had access to email. More research is needed with diverse groups of carers to determine if findings are similar. The methodology also involved focus group interviews, which did not allow for in-depth individual understanding of participants’ experiences. Focus groups are useful when discussing difficult topics, such as the carers’ experience, as some participants feel more comfortable in a group setting. However, even the experienced moderators could not ensure all participants were given the opportunity to fully describe their experience. More research is needed to gain an in-depth understanding of the carers’ experiences using individual interviews.

## Conclusion

The findings of this study describe how family carers deal with life-altering changes by building a new life and how carers could benefit from support in this process. Factors that supported them in building a new life also provide areas for future interventions. These included: a) understanding dementia, b) support from others (staff, family and friends), c) connecting with resources, and d) being included in care decisions. In some LTC settings improvements in care may be needed before meaningful relationships and sharing of care between staff and carers can be established. The findings also underscore how the quality of care and the environment in which the person with dementia resides are key factors that should be considered in future research with carers.

## Data Availability

The datasets generated and/or analysed during the current study are not publicly available as we did not obtain consent for this from participants. However, the de-identified/anonymized data are available from the corresponding author (Dr. Wendy Duggleby at wendy.duggleby@ualberta.ca) on reasonable request.

## Abbreviations

FG: Focus Group
LTC: Long Term Care
